# Transcriptomic and barrier responses of human airway epithelial cells exposed to cannabis smoke

**DOI:** 10.14814/phy2.14249

**Published:** 2019-10-23

**Authors:** Jennifer A. Aguiar, Ryan D. Huff, Wayne Tse, Martin R. Stämpfli, Brendan J. McConkey, Andrew C. Doxey, Jeremy A. Hirota

**Affiliations:** ^1^ Department of Biology University of Waterloo Waterloo Ontario Canada; ^2^ Division of Respiratory Medicine Department of Medicine University of British Columbia Vancouver British Columbia Canada; ^3^ Firestone Institute for Respiratory Health – Division of Respirology Department of Medicine McMaster University Hamilton Ontario

**Keywords:** Marijuana, Calu‐3 cells, Transepithelial electrical resistance, oxidative stress, interferon stimulated genes

## Abstract

Globally, many jurisdictions are legalizing or decriminalizing cannabis, creating a potential public health issue that would benefit from experimental evidence to inform policy, government regulations, and user practices. Tobacco smoke exposure science has created a body of knowledge that demonstrates the conclusive negative impacts on respiratory health; similar knowledge remains to be established for cannabis. To address this unmet need, we performed *in vitro* functional and transcriptomic experiments with a human airway epithelial cell line (Calu‐3) exposed to cannabis smoke, with tobacco smoke as a positive control. Demonstrating the validity of our *in vitro* model, tobacco smoke induced gene expression profiles that were significantly correlated with gene expression profiles from published tobacco exposure datasets from bronchial brushings and primary human airway epithelial cell cultures. Applying our model to cannabis smoke, we demonstrate that cannabis smoke induced functional and transcriptional responses that overlapped with tobacco smoke. Ontology and pathway analysis revealed that cannabis smoke induced DNA replication and oxidative stress responses. Functionally, cannabis smoke impaired epithelial cell barrier function, antiviral responses, and increased inflammatory mediator production. Our study reveals striking similarities between cannabis and tobacco smoke exposure on impairing barrier function, suppressing antiviral pathways, potentiating of pro‐inflammatory mediators, and inducing oncogenic and oxidative stress gene expression signatures. Collectively our data suggest that cannabis smoke exposure is not innocuous and may possess many of the deleterious properties of tobacco smoke, warranting additional studies to support public policy, government regulations, and user practices.

## Introduction

The United Nations World Drug Report estimates over 180 million global cannabis users (World Heatlh Organization ‐ World Drug Report, 2018). Decriminalization and legalization of cannabis in several global jurisdictions and greater access to medicinal cannabis may lead to increased use of cannabis products. In Canada, combustion is the most common form of cannabis consumption use as reported by 94 and 89% of users in the 2017 and 2018 Canadian Cannabis Surveys respectively (Canada H, 2017; Canada H, 2018). The negative effects of tobacco smoke on the lung and its airway epithelium are universally accepted (Auerbach et al., 1979; Jha et al., 2013; Thun et al., 2013). In contrast, the effects of cannabis smoke on human lung health are less clear (reviewed in Tashkin 2013) and data are needed to effectively inform policy, government regulations, and user practices.

Inhalation of cannabis smoke delivers pharmacologically active ingredients to the lung, including tetrahydrocannabinol (THC) and cannabidiol (CBD), as well as combustion products such as polycyclic aromatic hydrocarbons (Moir et al., 2008). Repeated cannabis smoke exposure is associated with higher incidence of coughing and shortness of breath relative to nonsmokers (Tashkin et al., 1987; Wu et al., 1988; Aldington et al., 2007; Tan et al., 2009); symptoms that are shared with tobacco smoking. Despite these reports there remains a paucity of observational and mechanistic cannabis smoke exposure studies and models that can inform our understanding of potential public health issues.

The lungs are in constant contact with harmful environmental agents such as viruses and bacteria yet we rarely show signs of infection (Parker and Prince, 2011; Huff et al., 2019). Minimized infection is the result of innate and adaptive immune processes that include the physical barrier and immunological functions of the airway epithelium. Protection rendered by the airway epithelium can be compromised by tobacco smoke (Hudy et al., 2010; Eddleston et al., 2011; Rider et al., 2011; Amatngalim et al., 2016), leading to increased susceptibility to infections and potential for host pathology. Whether cannabis smoke exposure similarly impacts airway epithelial cell function and immunity relevant in pathogen defence remains to be determined.

To address the above issues, we performed a series of *in vitro* functional and transcriptomic experiments with a human airway epithelial cell line (Calu‐3) exposed to cannabis smoke, with tobacco smoke used as a positive control. To model cannabis smoke exposure, we used smoke conditioned media methods that have been validated for tobacco combustion experiments (Wirtz and Schmidt, 1996; Bernhard et al., 2004; Bauer et al., 2008; Hudy et al., 2010; Hudy and Proud, 2013; Hudy et al., 2014; Amatngalim et al., 2016; Jamieson et al., 2019). Comparison of differential gene expression patterns from our tobacco smoke conditioned media experiments in Calu‐3 cells to bronchial brushings from human tobacco smokers (Harvey et al., 2007; Tilley et al., 2011) and air‐liquid interface cultures of primary epithelial cells exposed to mainstream tobacco smoke (Mathis et al., 2013; Haswell et al., 2017; Haswell et al., 2018) revealed overlap, validating the relevance of our model. Using cannabis smoke conditioned media, we observed functional and transcriptional responses that were shared with tobacco smoke. Gene expression pathway analysis revealed that cannabis smoke induced DNA replication and oxidative stress responses. Functionally, cannabis smoke impaired epithelial cell barrier function, antiviral responses, and elevated inflammation. Broadly speaking, our data demonstrate that cannabis smoke exposure is not innocuous and induces transcriptional and functional responses in human airway epithelial cells that may impact lung health similar to tobacco smoke.

## Materials and Methods

### Preparation of cannabis and tobacco smoke extracts

Cannabis smoke extract (CSE) and tobacco smoke extract (TSE) conditioned media were prepared according to previously published methods (Rider et al., 2011). For generation of the TSE, a Kentucky Research Grade Cigarette (Lot: 3R4F – cellulose acetate filter, ~0.7g of dried tobacco leaves) was used. For generation of the CSE, cannabis from Dr. Jonathan Page (University of British Columbia, Vancouver, British Columbia, Canada) (13% THCA strain (w/w), with 0.18% THC, 0.35% THCVA, and 0.18% CBGA, ~0.7g dried cannabis) rolled with cardboard filters was used. To prepare the smoke‐conditioned media, either 1 cannabis cigarette or 1 tobacco cigarette was smoked into 4ml of HEPES buffered Eagle’s Minimal Essential Medium (EMEM). Smoke extracts were filtered using a 0.22μm filter. Extracts were standardized by measuring absorbance and diluting with fresh medium to reach a desired dilution (OD260nm = 0.4045*dilution factor, 10% dilution = 0.04045 OD260nm). A single batch of CSE and TSE was generated, aliquoted, and stored at −80°C and used for all subsequent experiments. For RNA‐sequencing experiments, 10% CSE and TSE were used. For concentration‐response studies, 0.625, 1.25, 2.5, 5, 10, and 20% CSE and TSE were used.

### Epithelial cell culture and drugs

Calu‐3 cells obtained from ATCC (Manassas, Virginia, USA) were grown in EMEM with 10mM HEPES, 10% fetal bovine serum (FBS), and antibiotic‐antimycotic, and used between passages 10–20. For exposure experiments, 1 × 10^6^ or 2 × 10^5^ Calu‐3 cells were seeded onto either 4.7 cm^2^ or 0.3 cm^2^ polyester Transwell® permeable supports respectively, with a pore size of 0.4 μm. Cells were grown for 20 days to promote cell polarization, with media on both apical and basal sides (Stentebjerg‐Andersen et al., 2011). FBS was removed from Calu‐3 cultures 24 h prior to the start of the exposures. For exposures, fresh FBS‐free EMEM culture media was added to basal chambers and CSE or TSE media diluted with fresh FBS‐free EMEM culture media was added to the apical chambers for 24 h.

### Cell viability assay

Cell viability was assessed using a Pierce™ LDH Cytotoxicity Assay Kit (ThermoFisher, Mississauga, Ontario, Canada) according to the manufacturer’s instructions.

### Barrier function assessment

Transepithelial Electrical Resistance (TEER) was measured using a Millicell ERS‐2 Voltohmmeter (EMD Millipore, Etobicoke, Ontario, Canada). Resistance was measured just prior to exposure as well as 24 h post‐exposure and multiplied by the growth area of the inserts (Ohms*cm^2^).

### Cytokine Assays

Cell supernatants collected from the apical side of the culture system were analyzed by multiplexed laser bead assays using the 42‐plex human cytokine/chemokine protein arrays (Eve Technologies, Calgary, Alberta, Canada).

### RNA‐sequencing analysis

Total RNA was extracted using an RNeasy Plus Kit (Qiagen, Toronto, Ontario, Canada). cDNA was prepared at The Centre for Applied Genomics at the Hospital for Sick Children (Toronto, Ontario, Canada). Samples were sequenced on the Illumina HiSeq 2500 instrument with 125 bp paired‐end reads to a minimum depth of 30 million reads per sample. Reads were de‐multiplexed and trimmed and BCL files generated from the Illumina sequencer were converted to FASTQ files.

After quality control using FastQC (v.0.11.7) and Prinseq (v.0.20.4), sequences were aligned to the human reference genome (hg19) using HISAT2 (v.2.1.0) and assembled into full transcriptomes using StringTie (v.1.3.3b). Samtools (v.1.9) was used to convert and sort HISAT2 output into sorted bam files for use by StringTie. StringTie was also used to calculate transcript abundances for downstream differential expression analysis using the Ballgown package in R (v. 3.4.3) which provides *p* values, FDR‐adjusted *P* values (*q* values), and fold change values for all genes in each comparison. A snakemake‐based pipeline called hppRNA (v.1.3.3) was used to combine the above steps into a streamlined work‐flow.

### Validation of cell culture and exposure methods

To validate the use of Calu‐3 cells exposed to smoke extract‐conditioned media *in vitro*, four independent tobacco smoke exposure datasets were compared to the results from the TSE versus Control differential expression analysis. Two microarray datasets (GSE4498 and GSE11784) and two high‐throughput RNA‐sequencing datasets (SRP096285 and SRP126155) were used for validation. GSE4498 and GSE11784 datasets consist of gene expression data obtained from microarray analysis of mainstream tobacco cigarette smoke‐exposed airway epithelial cells compared to cells from healthy individuals (bronchial brushings). SRP096285 and SRP126155 datasets consist of nasal epithelial cells cultured on Transwells and exposed to either 3R4F reference tobacco cigarette smoke extract‐conditioned media or air. To determine the correlation between either of these datasets and our own dataset, a list of differentially expressed genes from the deposited dataset (adjusted *P* < 0.05) was compared to the differentially expressed genes from our TSE versus Control dataset (adjusted *P* < 0.05). The intersection of gene names from both lists was determined and these genes were plotted and their correlation was determined using Pearson’s r with *p*‐value reported. Significance of the gene overlap was determined by a hypergeometric test in R.

### Functional enrichment and pathway analyses

Lists of significantly differentially expressed genes were identified for CSE versus control and TSE versus control (as determined by Ballgown, FDR *q*‐value < 0.05) and genes present in both comparisons were submitted to EnrichR to identify enriched pathways and functional ontologies (Chen et al., 2013). Terms were ranked within ontologies by EnrichR’s combined score (log(*p* value) x z‐score of the deviation from the expected rank). Expected rank (FDR adjusted *P* value) was calculated by EnrichR by running the Fisher exact test for random gene sets in order to compute a mean rank and standard deviation from the expected rank for each term in the gene set library.

### Statistical analysis

Significant changes in cell viability, TEER, and cytokines were identified through permutation ANOVA followed by Tukey Honest Significant Difference (HSD) post‐hoc test using the “lmPerm” package in R (v. 3.4.3). Significant differences between transcriptional expression profiles were identified through ANOSIM using the “vegan” package in R (v.3.4.3). For all analyses, differences were considered statistically significant when FDR adjusted *p* values are less than 0.05, or equivalently with a false‐discovery rate of 5%. For all experiments, four independent biological replicates derived from distinct Calu‐3 stocks were performed (*n* = 4).

## Results

### *In vitro* cannabis smoke exposure‐induced changes in gene expression overlap with tobacco smoke

For our experiments, the transcriptomic changes induced by tobacco smoke were used as positive control stimulus to determine the relative impact of cannabis smoke exposure.

**Figure 1:**
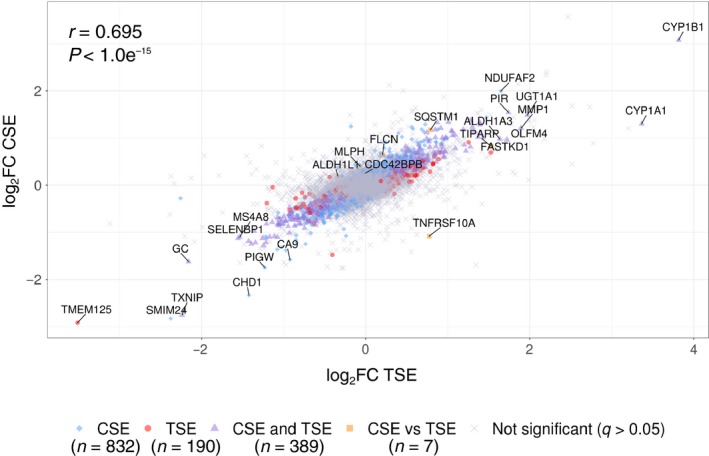
Cannabis and tobacco smoke extract‐induced transcriptomic changes. Correlation of differential gene expression profiles (log_2_FoldChange(FC)) versus control, comparing cannabis and tobacco exposures, as determined by Pearson’s correlation. Significantly differentially expressed genes in cannabis smoke extract (CSE) only (blue diamonds, *n* = 832), tobacco smoke extract (TSE) only (red dots, *n* = 190), and shared between CSE and TSE (purple triangles, *n* = 389) are highlighted. Only seven genes were identified as significantly differentially expressed between CSE and TSE (orange squares, *n* = 7).

Transcriptomic analysis of differentially expressed genes following cannabis or tobacco smoke exposure for 24 h at 10% dilution revealed a highly significant correlation (*r* = 0.695, *P* < 1.0*10^−15^) and overlap of shared changes in gene expression (Fig. 1, *n* = 389, purple triangles).

Enriched pathways and functional ontologies were explored based on gene expression patterns using the bioinformatic tool, EnrichR (Chen et al., 2013) (data not shown). Significantly differentially expressed genes that overlapped between CSE‐exposed and TSE‐exposed (*n* = 389, purple triangles) cells were analyzed. Analysis of these shared significantly differentially expressed genes revealed increased expression of genes involved in the NRF2 and aryl hydrocarbon receptor pathways that are both involved in responding to oxidative stress and xenobiotic molecules. Moreover, the two genes most upregulated by both smoke exposures were aryl hydrocarbon receptor induced genes *CYP1A1* and *CYP1B1*. We also observed increases in genes involved with cell replication pathways, cell cycle activating transcription factors E2F1 and MYC, altered barrier function, and lowered antiviral responses in smoke‐exposed cells (see Figs. 3, 4).

**Figure 2:**
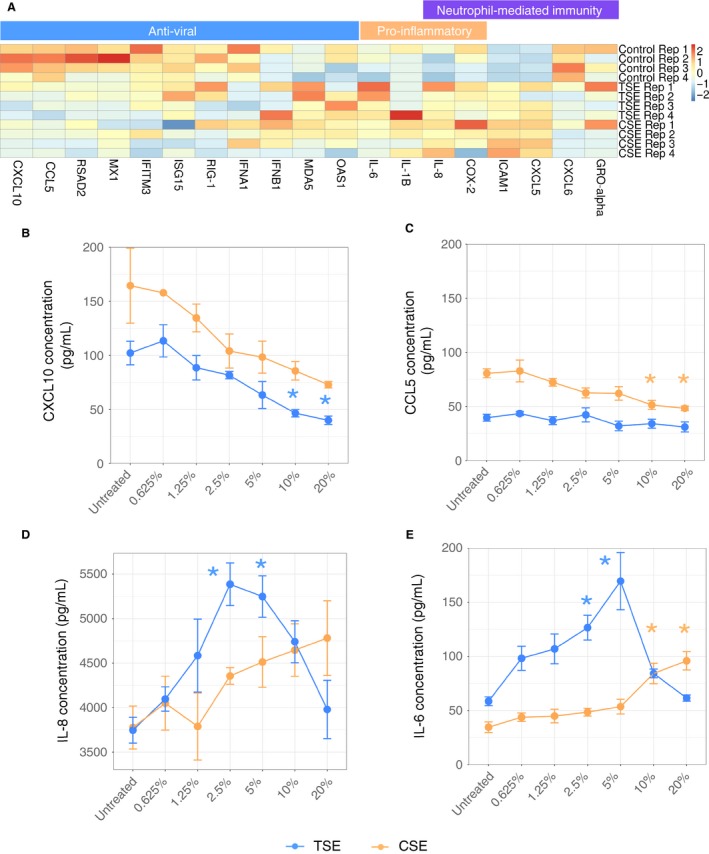
Transcriptomic correlations of *in vitro* tobacco smoke exposure in Calu‐3 cells to human smokers and primary airway epithelial cells. Correlation of Calu‐3 cell differential gene expression profile (log2FoldChange (FC)) following exposure to tobacco smoke extract (TSE) and differential gene expression profiles between healthy controls and lifetime smokers in the publicly available datasets (A) GSE4498 and (B) GSE11784. Genes that are identified in both datasets and exhibit statistically significant differences in expression are included. Correlations with differential gene expression profiles from primary human airway epithelial cells grown under air‐liquid interface culture conditions and exposed to main‐stream tobacco smoke using the publicly available datasets (C) SRP096285 and (D) SRP126155.

**Figure 3:**
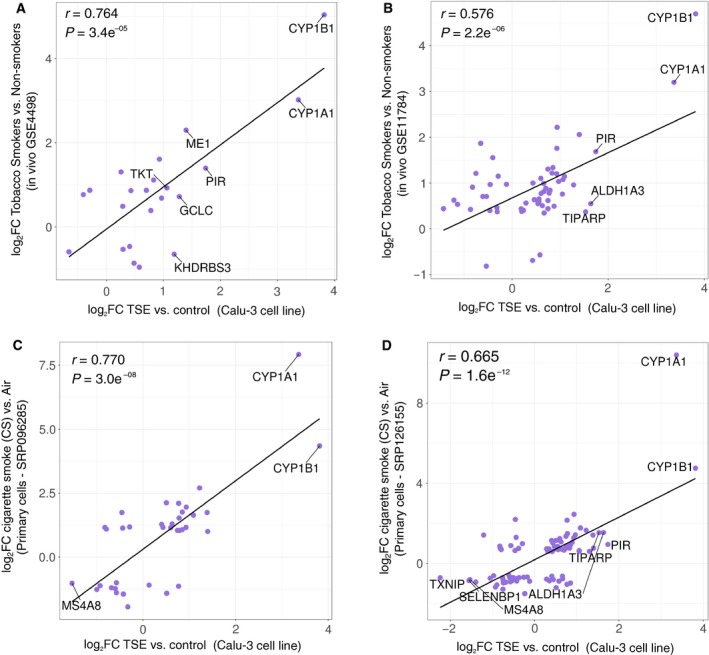
Impact of cannabis smoke exposure on epithelial cell barrier function and transcriptomic profile. Genes involved in (A) airway epithelial repair and remodeling or epithelial junctions were curated from the RNA‐sequencing dataset performed at 10% CSE or TSE. The expression for each gene is presented for all experimental replicates, with expression for each replicate being scaled by the gene. Calu‐3 cells were exposed to increasing concentrations of CSE (orange) or TSE (blue) (control, 0.625%, 1.25%, 2.5%, 5%, 10%, and 20%) for 24h with outcome measurements of (B) cell viability assessed by lactate dehydrogenase (LDH) assay, (C) transepithelial electrical resistance – TEER (ohms*cm^2^), (D) transforming growth factor‐alpha (TGF‐α) (pg/mL), and (E) platelet derived growth factor‐AA (PDGF‐AA) (pg/mL). *=*P* < 0.05 relative to control untreated ‐ Tukey HSD. Error bars represent standard deviation (*n* = 4).

**Figure 4:**
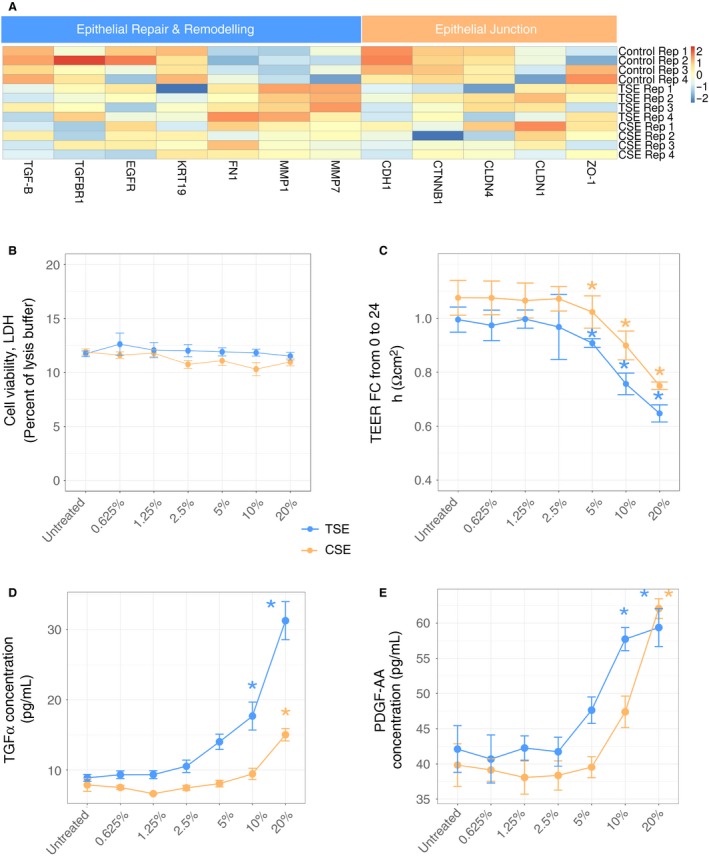
Impact of cannabis smoke exposure on epithelial cell antiviral and inflammatory expression and transcriptomic profile. Genes involved in (A) airway epithelial anti‐viral, pro‐inflammatory, or neutrophil mediated immunity were curated from the RNA‐sequencing dataset performed at 10% CSE or TSE. The expression for each gene is presented for all experimental replicates, with expression for each replicate being scaled by the gene. Calu‐3 cells were exposed to increasing concentrations of CSE (orange) or TSE (blue) (control, 0.625%, 1.25%, 2.5%, 5%, 10%, and 20%) for 24h with outcome measurements of (B) interferon gamma induced protein‐10 (CXCL10) (pg/mL), (C) regulated on activation, normal T cell expressed and secreted (CCL5) (pg/mL), (D) IL‐8 (pg/mL), and (E) IL‐6 (pg/mL). *=*P* < 0.05 relative to control untreated ‐ Tukey HSD. Error bars represent standard deviation (*n* = 4).

Minor differences in responses between CSE and TSE reveals that cannabis smoke exposure is able to induce selective changes in gene expression that are not altered with tobacco smoke (Fig. 1, blue diamonds, *n* = 832) or were different from tobacco smoke (Fig. 1, orange squares, *n* = 7). Only seven genes (*ALDH1L1*, *CDC42B2B*, *MLPH*, *SQSTM1*, *TIPARP*, *TNFRSF10A*, and *FCLN*) were differentially expressed between CSE and TSE exposure. However, these genes may be of interest in the context of airway epithelial cell biology following smoke exposure. For instance, *TNFRSF10A* (Death Receptor 4, *DR4*) was significantly down‐regulated in CSE relative to TSE exposed cells (4.8 fold change).

Collectively, these results demonstrate that cannabis smoke exposure impacts transcriptional responses in airway epithelial cells consistent with an oxidative stress phenotype, cell replication, and altered innate immunity.

### *In vitro* tobacco smoke exposure of Calu‐3 cells recapitulates differential gene expression patterns observed in human smokers and primary airway epithelial cells

*In vitro* tobacco smoke exposure models have been extensively used with many experimental variations including cell type (cell line vs. primary), culture format (submerged monolayer vs. air‐liquid interface), or exposure format (smoke conditioned media vs. mainstream smoke). We selected Calu‐3 cells grown under submerged monolayer conditions as an *in vitro* model for studying the transcriptomic effects of cannabis smoke exposure (Kreft et al., 2015).

To determine the ability of this model to recapitulate biologically relevant gene expression patterns, we generated an RNA‐sequencing transcriptomic dataset of Calu‐3 cells following 24h tobacco smoke exposure (10% TSE) and compared it with previous transcriptomic datasets derived from (1) airway epithelial cells isolated from bronchial brushings of lifetime tobacco smokers; (2) primary human airway epithelial cells grown under air‐liquid interface culture conditions and acutely exposed to main stream tobacco smoke (Harvey et al., 2007; Tilley et al., 2011; Haswell et al., 2017; Haswell et al., 2018).

We observed correlation (*r* = 0.764, *P* = 3.4*10^−5^ and *r = *0.576, *P* = 2.2*10^−6^) of differentially expressed genes between the transcriptomes of Calu‐3 cells exposed to tobacco smoke conditioned media and bronchial brushings obtained by tobacco smokers in two independent datasets (GSE4498 and GSE11784) (Harvey et al., 2007; Tilley et al., 2011) (Fig. 2A–B). Furthermore, expression levels of differentially expressed genes were also correlated (*r* = 0.770, *P* = 3.0*10^−8^ and *r = *0.665, *P* = 1.6*10^‐12^) between tobacco smoke exposure Calu‐3 cells and air‐liquid interface cultures of primary human airway epithelial cells exposed to mainstream tobacco smoke in two independent datasets (SRP096285 and SRP126155)(Haswell et al., 2017; Haswell et al., 2018)(Fig. 2C–D). A hypergeometric test shows significant overlap of differentially expressed genes between the transcriptomes of Calu‐3 cells exposed to tobacco smoke conditioned media and bronchial brushings from both microarray datasets (GSE4498 and GSE11784, Figure S1A–B). Significant overlap of differentially expressed genes between the transcriptome of Calu‐3 cells exposed to tobacco smoke conditioned media and SRP126155 but not SRP096285 datasets containing air‐liquid interface cultures (Figure S1C–D). Collectively, these results support the validity of using Calu‐3 cells under submerged monolayer culture conditions with smoke conditioned media to model and interrogate human airway epithelial cell responses to cannabis.

### *In vitro* cannabis smoke exposure impairs epithelial cell barrier function and related cytokine production without impacting viability

Previous studies have implicated oxidative stress in disrupting epithelial barrier function (Boardman et al., 2004), therefore our transcriptomic analysis demonstrating oxidative stress suggested that barrier function could be compromised in cannabis smoke exposed cells.

Genes associated with epithelial repair and remodeling were curated from our RNA‐seq analysis and plotted in a heatmap from our transcriptomic dataset (Fig. 3A) including *TGFB* (transforming growth factor‐beta), *TGFBR1* (transforming growth factor‐beta receptor 1), *EGFR* (epidermal growth factor receptor), *KRT19* (cytokeratin 19), *FN1* (fibronectin), *MMP1* (matrix metalloproteinase 1), and *MMP7* (matrix metalloproteinase 7) (Fig. 3A). In parallel, an additional set of genes associated with epithelial cell barrier function were curated including *CDH1* (E‐Cadherin), *CTNB1* (β‐catenin), *CLDN4* (Claudin 4), *CLDN1* (Claudin‐1), and *ZO‐1* (Zonus Occludin‐1). Cannabis smoke exposure results in both the upregulation (*FN1, MMP1*) and downregulation (*TGFB1, CDH1, CTNNB1*) of genes important in epithelial repair, remodeling, and barrier function (adjusted *P* < 0.05). Similar directions of trends were observed for tobacco smoke‐induced changes in these genes.

To interrogate transcriptomic changes in the context of cell viability, trans‐epithelial electrical resistance (TEER), and cytokine production at the protein level, a concentration response of cannabis smoke exposure was performed.

No changes in cell viability were observed at any concentration of cannabis smoke conditioned media, suggesting cell death was minimally impacted by cannabis smoke (Fig. 3B). In the absence of any cell death, cannabis smoke exposure resulted in a concentration‐dependent decrease in TEER at 5, 10, and 20% smoke conditioned media (Fig. 3C). The viability and barrier function results were comparable with tobacco smoke exposure.

We next explored the functional consequences of cannabis smoke exposure on cytokine and growth factor protein production important in epithelial cell barrier function. Cannabis smoke exposure resulted in a concentration dependent increase in TGF‐α and PDGF‐BB at 20% smoke conditioned media (Fig. 3D–E).

### *In vitro* cannabis smoke exposure attenuates epithelial cell antiviral cytokine responses and induces pro‐inflammatory cytokine production

Transcriptomic changes induced by cannabis smoke exposure suggested that antiviral and pro‐inflammatory immune responses could be attenuated and induced respectively.

Genes associated with antiviral immunity and pro‐inflammatory responses were curated from our RNA‐seq analysis and plotted in a heatmap from our transcriptomic dataset (Fig. 4A) including *CXCL10*, *CCL5*, interferon stimulatory genes, *IL‐6, IL‐1β, IL‐8,* and several chemokine receptors (Fig. 4A). Cannabis smoke exposure results in both the upregulation (*IL‐8, ICAM‐1, CXCL5, CXCL6)* and downregulation (*CXCL10, CCL5, RSAD2, IFITM3*) of genes important in antiviral immunity and pro‐inflammatory responses (adjusted *P* < 0.05). Similar directions of trends were observed for tobacco smoke‐induced changes in these curated genes.

Antiviral and pro‐inflammatory cytokine transcriptomic changes were analyzed at the protein level in the concentration‐response study samples. Cannabis smoke exposure resulted in a concentration dependent trend for a reduction in resolution also bad on this figure CXCL10 and a significant reduction in CCL5 at 10 and 20% smoke conditioned media (Fig. 4B–C). These trends were comparable with tobacco smoke exposure.

We next explored the functional consequences of cannabis smoke exposure on pro‐inflammatory cytokine production. Cannabis smoke exposure resulted in a concentration dependent trend for increased IL‐8 and a significant increase in IL‐6 at 10 and 20% smoke conditioned media (Fig. 4D–E). These trends were also conserved with tobacco smoke exposure.

## Discussion

Global cannabis use is an important public health issue that would benefit from experimental evidence to inform policy, government regulations, and user practices. Analyses that interrogate cannabis in parallel with a positive control of tobacco smoke will help provide clinically relevant evidence and context (Auerbach et al., 1979; Tashkin et al., 1987; Wu et al., 1988; Jha et al., 2013; Thun et al., 2013). Our *in vitro* functional and transcriptomic experiments with Calu‐3 human airway epithelial cells exposed to cannabis smoke demonstrate changes in gene expression signatures related to DNA replication and oxidative stress responses, an impairment of barrier function and antiviral immune responses, and an augmented pro‐inflammatory cytokine profile. Importantly, all cannabis‐induced responses were observed in tandem with our positive control of tobacco smoke, suggesting potential parallel implications for lung health.

*In vitro* experiments using human airway epithelial cell lines and primary cell samples under different growth conditions have been essential methods in tobacco smoke exposure science (Wirtz and Schmidt, 1996; Bernhard et al., 2004; Bauer et al., 2008; Hudy et al., 2010; Hudy and Proud, 2013; Mathis et al., 2013; Hudy et al., 2014; Haswell et al., 2017; Haswell et al., 2018; Jamieson et al., 2019), while no such diversity and baseline of data currently exists for cannabis smoke exposure science. Potential exists for differences in experiment design (e.g. submerged monolayer cell line exposed to smoke‐conditioned media vs. air‐liquid interface primary airway epithelial cell exposed to mainstream smoke), which may confound and limit translation of experimental results for any smoke exposure. To assess the validity of our *in vitro* model of Calu‐3 cells exposed to smoke conditioned media, we compared the differentially expressed genes from our tobacco exposure experiments with publicly available datasets from gold standard bronchial brushings in addition to an air‐liquid interface model of primary human airway epithelial cell cultures. Differential gene expression analyses demonstrate significant correlation between our *in vitro* model of Calu‐3 cells with *in situ* bronchial brushings and *in vitro* air‐liquid interface cultures of primary human airway epithelial cells. Importantly, significant correlation existed between our model and *in situ* profiles and more complex *in vitro* models, despite the use of the Calu‐3 cell line (relative to primary cells) and smoke‐conditioned media (relative to main‐stream smoke). The results from our cannabis smoke exposure studies are therefore potentially reflective of the *in situ* condition, although this should be validated with clinical studies.

The function of the airway epithelium is to provide the lung a physical and immunological barrier to the environment (Parker and Prince, 2011; Huff et al., 2019). Any perturbation in the airway epithelium may lead to host susceptibility to infection and lung pathology or disease development. Our data demonstrate that cannabis smoke is able to induce mild impacts on barrier function, measured by TEER, without impacting cell viability. The mechanism(s) by which TEER is reduced by cannabis smoke were not determined in our study, but in the absence of changes in cell viability it is possible that cell‐cell junctions could have been disrupted as has been reported for tobacco smoke (Schamberger et al., 2014), possibly by oxidative stress (Boardman et al., 2004). The down‐regulation of E‐cadherin (*CDH1*) by cannabis smoke has been previously observed in lung cells exposed to tobacco smoke and associated with the induction of epithelial‐to‐mesenchymal transition, migration, and invasion phenotypes (Nagathihalli et al., 2012) that could be important in the context of lung health of the cannabis smoker. Disruption of E‐cadherin may contribute to β‐catenin shuttling from membrane junction sites to transcriptional locations in the nucleus to facilitate gene expression associated with repair (Moheimani et al., 2015). Importantly, aberrant β‐catenin signaling in cells is associated with oncogenic gene expression signatures (Emami et al., 2004). Independent of any aberrant transcriptional regulation resulting from disrupted epithelial barrier function; a reduced impedance of the physical barrier of the airway epithelium offers easier access to the lung for opportunistic insults from the outside world.

To complement the mechanical barrier of the lung, the airway epithelium is able to produce and host‐defence peptides and antiviral mediators to protect from pathogens (Parker and Prince, 2011; Huff et al., 2019). Tobacco smoke has been reported to compromise the ability of airway epithelial cells to effectively control both bacterial and viral insults (Hudy et al., 2010; Eddleston et al., 2011; Rider et al., 2011; Amatngalim et al., 2016). Our data demonstrating striking similarities in the epithelial immune profile in response to cannabis and tobacco, suggests that the former will impact host defences. Tobacco has been demonstrated to impact host defence peptide induction in airway epithelial cells by nontypeable *Haemophilus influenzae* with a concomitant increase in IL‐8 expression (Amatngalim et al., 2017). Our experimental dataset in this study did not include a pathogen challenge, precluding our ability confirm the tobacco smoke induced suppression of host defence peptides or to extend the results to cannabis smoke. In the context of viral exposures, type I interferons (IFNs) are capable of rapidly inducing interferon stimulated genes (ISGs) through the type I IFN receptor to help tackle various components of virus replication, assembly and budding (Goubau et al., 2013). Tobacco smoke has been shown to impact antiviral immunity in airway epithelial cells in response to human rhinovirus‐16, with a reduction in CXCL10 and CCL5 ( Eddleston et al., 2011). Tobacco smoke‐induced reduction in CXCL10 and CCL5 is associated with greater rhinovirus production. Tobacco smoke has also been demonstrated to impair airway epithelial cell antiviral immunity mediated by IFN‐γ in response to respiratory syncytial virus exposure (Modestou et al., 2010), which in turn could impact CXCL10 and CCL5 production (Pawliczak et al., 2005). Our cytokine and transcriptomic data confirm the tobacco smoke impairment of CXCL10 and CCL5 and antiviral ontologies and show that this is conserved for cannabis smoke. We further demonstrate using our transcriptomics dataset that a diverse selection of ISGs were also attenuated with both cannabis and tobacco, consolidating a common phenotype between both smoke exposures. Future co‐culture experiments with pathogen challenges would help reveal the complex interaction between cannabis and tobacco smoke induced immune responses in the epithelium and down‐stream immune cell phenotype and function. Collectively, although our experimental designs lack the mechanistic linkage between cannabis exposure and increased susceptibility to viral or bacterial infections, our data strongly mirror those for tobacco, which has been mechanistically linked to compromised host immunity to pathogens.

Extensive evidence exists that tobacco and biomass smoke exposure, the latter generated from dried wood, animal dung, or charcoal, are risk factors for the development of chronic bronchitis, emphysema, and lung cancers (Gordon et al., 2014; Kurt et al., 2016; Kc et al., 2018; Huff et al., 2019). In contrast, the existing evidence suggests that repeated cannabis smoke exposure results in a chronic bronchitis phenotype with little evidence of emphysema (Tashkin et al., 1987; Wu et al., 1988; Aldington et al., 2007; Tan et al., 2009). Furthermore, unlike tobacco and biomass exposure, which are accompanied by a dose‐dependent risk for development of lung cancers, a similar relationship has not been observed for repeat cannabis users despite the presence of carcinogens in cannabis smoke (Zhang et al., 2015). Of particular note, we observed that cannabis smoke significantly upregulated the proto‐oncogene *MYC,* which has previously been observed in airway epithelial cells exposed to tobacco smoke (Lu et al., 2017) or benzo‐a‐pyrene, a known carcinogen that has been identified in both tobacco and cannabis smokes (Fields et al., 2004; Moir et al., 2008). Despite the potential for cannabis smoke to upregulate proto‐oncogenes, why cannabis smoke exposure is only tenuously linked to cancer (Tashkin, 2013) remains to be determined and should be explored in additional cohorts in jurisdictions where cannabis has been legalized.

Tobacco smoke exposure experiments have used standardized research source material to ensure experimental reproducibility and robustness. In contrast, cannabis experiments have not benefited from a widely accessible and chemically defined source material. For this reason, we decided to use a cannabis strain that was representative of that available in the medicinal cannabis market in Canada, that included 13% THCA (w/w), 0.18% THC, 0.35% THCVA, and 0.18% CBGA with no levels of CBD. Our results must therefore be interpreted based on this initial chemical composition and care should be taken to generalize that all cannabis strains will induce similar responses. Indeed, increasing evidence suggests that there may be complex interactions between THC and CBD via the CB1 and CB2 cannabinoid receptors that could impact the immunomodulatory functions of cannabis smoke (Srivastava et al., 1998; Pacher and Kunos, 2013; Boggs et al., 2018). Exposure studies could collectively benefit by reporting the chemical composition of the strain that was used to help facilitate interpretation of data generated.

Methods incorporating the use of smoke conditioned media have been used extensively for tobacco research (Wirtz and Schmidt, 1996; Bernhard et al., 2004; Bauer et al., 2008; Hudy et al., 2010; Hudy and Proud, 2013; Hudy et al., 2014; Amatngalim et al., 2016; Jamieson et al., 2019). Smoke conditioned media methods typically filter coarse particulates and extract water‐soluble components of smoke combustion. The reduction in compositional complexity of smoke conditioned media relative to mainstream smoke may be important, although our data and those of others (Mathis et al., 2013; Haswell et al., 2017; Haswell et al., 2018) demonstrate that major transcriptional changes are conserved in either model system (smoke conditioned media or mainstream smoke) and both are reflective of *in situ* human biology (Harvey et al., 2007; Tilley et al., 2011). Importantly, the presence or absence of filters in tobacco (cellulose acetate) and cannabis smoke (cardboard) exposure models should be considered in interpreting the present data and designing future experiments (Aufderheide et al., 2015). Collectively, these data validate smoke conditioned media models and suggest that they will provide insight into the impacts of cannabis smoke exposure on airway epithelial cell biology.

In conclusion, our data demonstrate striking similarities in the impacts of cannabis and tobacco smoke on airway epithelial cell barrier function, cytokine profile, and gene expression signatures. Despite the arrival of cannabis legalization, our data suggest that cannabis smoke exposure still poses a significant health risk and warrants ongoing study to build a body of evidence to support public policy, government regulations, and user practices.

## Conflict of Interest

None declared.

## Supporting information




**Figure S1:** Union of differentially expressed genes between our tobacco smoke exposure experiment in Calu‐3 cells and the genes differentially expressed in GSE4498, GSE11784, SRP096285, and SRP126155 calculated with a hypergeometric test in R.
