# Supplementary material for: Transcriptomic and barrier responses of human airway epithelial cells exposed to cannabis smoke
Source: Physiol Rep. 2019 Oct 23;7(20):e14249. doi: 10.14814/phy2.14249 (PMC6811686; doi:10.14814/phy2.14249)
Supplement: Supplementary file 1 — Figure S1: Union of differentially expressed genes between our tobacco smoke exposure experiment in Calu‐3 cells and the genes differentially expressed in GSE4498, GSE11784, SRP096285, and SRP126155 calculated with a hypergeometric test in R. [file PHY2-7-e14249-s001.pdf]

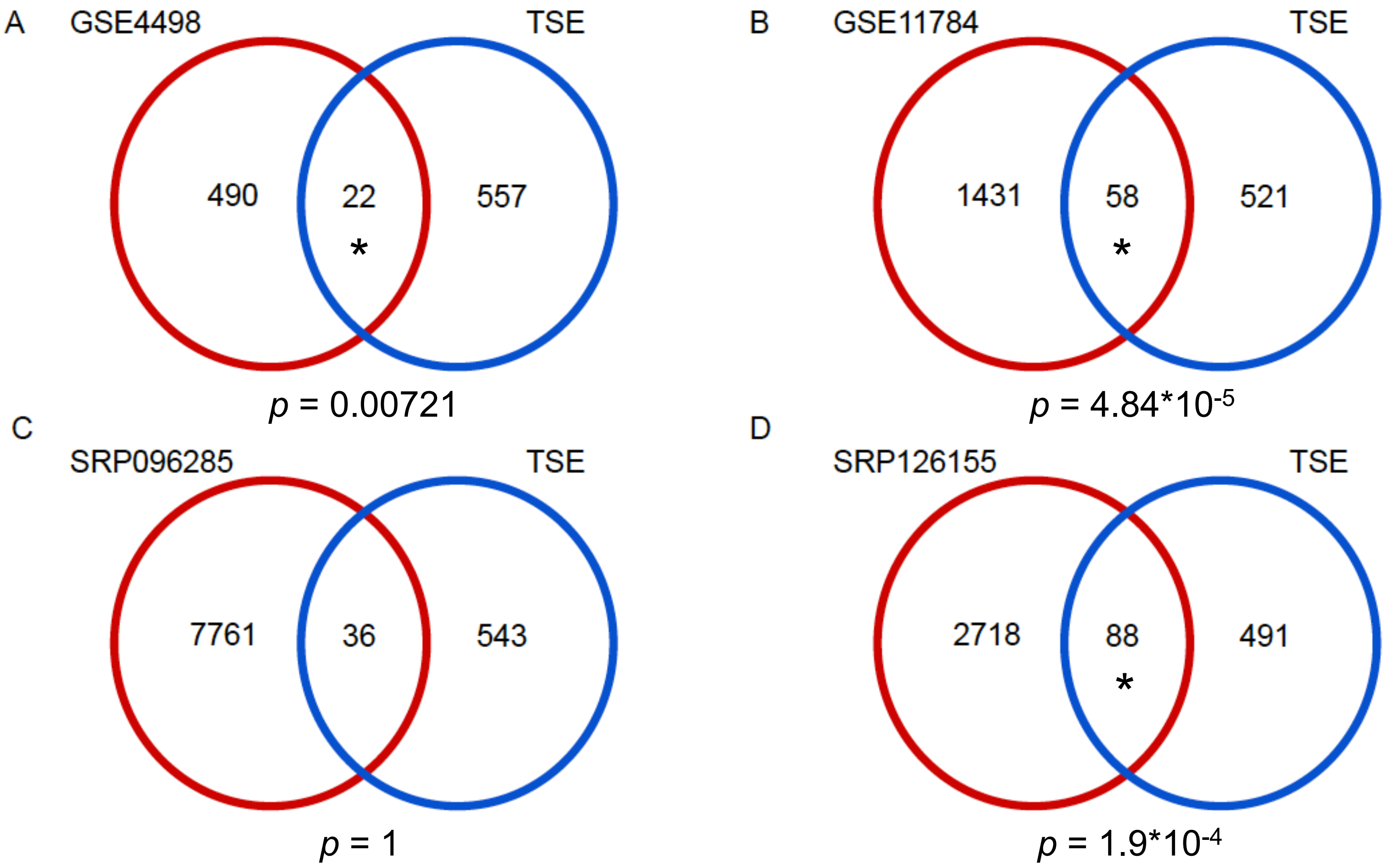

Supplement Figure 1: Union of differentially expressed genes between our tobacco smoke exposure experiment in Calu-3 cells and the genes differentially expressed in GSE4498, GSE11784, SRP096285, and SRP126155 calculated with a hypergeometric test in R.
